# Patient positioning in the proton radiotherapy era

**DOI:** 10.1186/1756-9966-29-47

**Published:** 2010-05-13

**Authors:** Salvatore Devicienti, Lidia Strigari, Marco D'Andrea, Marcello Benassi, Vincenzo Dimiccoli, Maurizio Portaluri

**Affiliations:** 1 Radiotherapy ASL Brindisi "A.Perrino" Hospital, Brindisi, Italy; 2 Laboratory of Medical Physics and Expert Systems, Regina Elena National Cancer Institute, Rome, Italy; 3 Technical division ITEL Telecomunicazioni s.r.l. Ruvo di Puglia (Ba), Italy; 4 Institute of Clinical Physiology - National Research Council (IFC-CNR), Pisa, Italy

## Abstract

The main hindrance to the diffusion of proton therapy facilities is the high cost for gantry installations. An alternative technical option is provided by fixed-beam treatment rooms, where the patient is rotated and translated in space with a robotic arm solution to enable beam incidence from various angles. The technological efforts based on robotic applications made up to now for patient positioning in proton beam facilities are described here, highlighting their limitations and perspectives.

## Review

There is currently an increasing interest in proton therapy in the world and the number of proton therapy facilities is rapidly increasing; mostly owing to the fact that physicians acknowledge that even the best current technique of X-ray therapy (intensity modulated proton therapy, IMRT) are still far from maximizing the therapeutic gain, i.e. increasing the local tumour control and decreasing the morbidity in healthy tissues. The concern about late effects for "low" doses to normal organs is particularly relevant in children. At the moment there are approximately 25 proton centres in operation worldwide and dozens of new ones are being planned. The aim of this work is to describe the most representative patient positioning solutions which are in clinical use in some proton radiotherapy centres and to comment on the advantages of robotic positioning in fixed beam delivery scenarios in terms of cost-effectiveness as compared to the moving gantry delivery solutions.

### Obstacles to the diffusion of proton therapy

The principal obstacle to the diffusion of proton therapy is the high cost for installation. Currently, proton-therapy is more expensive than photon-therapy and the high costs are mostly due to the beam delivery system. In 2003, Goitein and Jermann [1] estimated the relative costs of proton and photon therapy, concluding that, with some foreseeable improvements, the ratio of costs protons/photons was likely to be about 1.7. However, these estimates are probably outdated. Reimbursement rates currently allow the development and operation of proton-therapy facilities with a reasonable profit margin. In the future, it is likely, as these facilities reach full operational capacity that the reimbursement rates for proton-therapy treatment delivery will decrease as capital costs are spread among more patients.

One of the main issues in assessing the cost-effectiveness of proton-radiotherapy is the choice between moving gantries and fixed gantries with robotic patient positioning systems. In fact there are two types of beam lines in treatment rooms: isocentric gantries and fixed (usually horizontal) beam lines. In isocentric gantry rooms, the structure supports the beam line including large bending magnets that cause the beam to be bent first in any direction focusing on the target. The gantries, with their magnets and counterweights, using present technology, typically weigh from 120 to 190 tons. The rotating diameter of an isocentric gantry is typically 10 m or more, some smaller diameter gantries (i.e. compact gantries typically < 3 m) exist; however, depending upon the design they weigh even more. The entire gantry structure can be rotated in space around the patient so that the beam can be directed at the patient from a limited angle range (e.g. within a 180-degree rotation) or from any angle (within a 360-degree gantry rotation), depending on the technology. The largest isocentric gantries, because of their size, require a larger shielded vault up to 3 stories high.

In fixed-beam treatment rooms the beam is directed with an array of magnets to the nozzle which is fixed in space. Then, the patient is rotated and translated with a robotic system to enable beam incidence from various angles for optimal target coverage. Fixed-beam rooms are about three times smaller than gantry rooms; therefore the size of the volume to be shielded is significantly reduced. Fixed-beam rooms can be used for many treatment sites [2], although the full applicability for all tumour sites has not yet been investigated.

Goiten [3] argued that replacement of gantries by one or a few fixed beams in order to reduce the cost of a facility would be likely to result in sub-optimal treatments in a significant proportion of cases, but this depends on the kind of technology adopted for positioning.

Smith et al. [4] suggested some project solutions to improve efficiency with lower costs, such as: a) using treatment setup rooms outside the treatment room, which should improve the patient outcome, especially for paediatric patients who need to spend more time in the treatment setup room, also due to anaesthesia procedures; b) using faster, automated imaging techniques for patient positioning both outside and inside the treatment room; c) using robotics for transferring and positioning patients both outside and inside the treatment room, for moving imaging devices, and for handling treatment appliance.

### Patient positioning systems

In modern X-ray radiotherapy, patients can be positioned in the treatment room by automatic couches with 6 degrees of freedom (i.e. allowing translation and rotations). In isocentric gantry treatment rooms, the combination of gantry and couch movement provides greater flexibility in delivering multiple beams, from different directions, to optimize the dose distribution. Recently, robots have been introduced into particle therapy applications to be used for holding and positioning imaging systems or to replace traditional patient couches. Accuracy and reproducibility of these devices are very important in their design and development.

Moreover, lasers and imaging devices (x-ray tubes and image receptors) need to be included in each treatment and/or setup room. The lasers are used for initial patient set up (to get the patient close to the treatment position) and the imaging systems provide orthogonal (or in some cases three-dimensional) images of the patients to be compared with digitally planning images generated by treatment planning systems.

Modern technology could again improve the evaluation of correct patient or beam positioning. This could lead to new positioning and immobilization solutions for initial setup and for patient/organ motion management [5].

### The Midwest Proton Radiotherapy Institute (MPRI)

At MPRI (Bloomington, IN, USA) protons are produced in an accelerator and are transported by magnetic beam lines to one or more treatment rooms. The MPRI facility has three rooms. One of these has fixed horizontal beam lines, and the other two have gantries that rotate 360° around the isocentre. A novel positioning system has been designed based on commercial industrial robot arms with six degrees of freedom (three translational directions and three angles, pitch, roll and yaw) [6].

In the MPRI fixed beam room, a small robot (Motoman UP20) serves as a positioning platform for a radiographic panel used in image-guided patient positioning, and a larger one (Motoman UP200) positions patients on a bed or in a chair. In addition, the large robot serves as a crane for quick changes of the removable heavy brass collimation snouts between patient treatments, and for supporting and quickly positioning large devices, such as water phantoms, that are used outside of treatment for dosimetry and quality assurance measurements.

Industrial robots, such as the Motoman UP200, are designed for applications demanding very high precision, therefore, the speed and the acceleration of movements are strictly limited to guarantee patient safety and comfort. There are two distinct types of movements that are performed by the robot control software, i.e. large-scale moves along calculated paths, and small-scale jogs between nearby robot locations for making fine adjustments to the patient position.

During treatment, two radiotherapists are required to move either robot. One operating the controls, while the other standing next to the patient, to signal and prevent collisions. The controls of the patient positioning robot are operated from the software console. The Digital Radiography (DR) panel robot is a simpler system, operated with the commercially-supplied hand pendant. The use of a pull-down mechanism for the DR panel allows one to have the desired position repeatability of the UP20 robot, while keeping all the DR panel apparatus far from the patient whenever the robot is in motion. The patient's bed and chair are fitted with tilt sensors and accelerometers that inhibit robot motion in hardware via an emergency stop circuit in the controller unit. The accelerometers move at an acceleration of about 0.5 *g*, which corresponds to a light tap on the bed surface, and the tilt sensors allows up to 12° tilt from the level plane. The coupler that attaches the bed or chair to the robot is a standard industrial pneumatically-driven device, but it is supplemented by a manual locking mechanism that prevents the bed or chair from accidental decoupling. Joint limits on speed and acceleration are chosen by the clinical staff to be consistent with comfortable patient transport and can be set permanently in the robot controller.

### The Paul Scherrer Institute (PSI) remote positioning

The PSI delivery system currently in use, namely GANTRY 1, is build for remote positioning [7]. Before each fraction, patient fixation to the treatment table is performed in a dedicated treatment preparation room. The table is then moved, using a dedicated transporter, and coupled to the CT scanner (GE Hi-speed, Chalfont St. Giles, UK) for position verification. The transporter, the CT scanner and the treatment gantries coupling systems have been designed to guarantee a positioning accuracy within 1 mm and the coupling/decoupling of the table of both systems requires about 2 min.

Gantry and CT scanner isocenters are coincident to allow the same positioning accuracy. Once the table is coupled to the CT scanner, orthogonal scout images are taken and compared with the corresponding ones generated at the time of acquisition of the CT scan used for planning (acquired on the same CT scanner).

On the basis of the daily images, translational corrections to the table at the treatment gantry are calculated to minimize patient misalignment. After completing imaging and analysis procedures, the patient and table are uncoupled from the CT scanner and moved into the treatment room. The distance from CT to treatment gantry is approximately 20 m, requiring approximately 2 min for transportation.

Since there is a risk that the patient moves during transportation, scout images are periodically acquired after irradiation (usually every 10^th ^fraction), allowing an assessment of the extent of target movement and its consequences on the treatment dose delivery.

The new delivery system at PSI, named GANTRY 2, not yet in use, has a robotic couch with three degrees of freedom that can transport the patient between the beam gantry and a CT scanner placed in the treatment room. In this way patient fixation and verification are performed directly in the treatment room without an additional transportation system.

### The Centre de proton-therapie d'Orsay

In hadrontherapy centres that have only fixed horizontal beams (i.e. most carbon ions centres and first generation protons centres), the beam incidence angles remain technically limited, especially for treatment of children under general anaesthesia needing posterior-oblique (40 degrees or so) beams in the supine position. Therefore at Orsay a system allowing the child positioning on a 30° inclined (left or right) treatment table while keeping the child under general anaesthesia has been recently developed [8]. The supine position improves patient comfort and treatment quality and gives an easier approach to the anaesthetic team. The table is made of polystyrene (with a maximum beam attenuation of 3%), is 79 cm long and allows 10° recovery and 40° incidence angles. Regarding the contention system, an easy transportable device, low production costs and reproducible patient positioning, is necessary. The chosen solution at Orsay is a 3 cm thick, 60 cm wide and 137 cm long polystyrene plate placed on the treatment table. The plate can be moved for any kind of lateral beam (from the left or right), and has a fixation system for the thermoformed mask and straps for patient contention. A carbon insert has been placed into the polystyrene plate to mask positioning. This system is designed for use with every beam angle and patient size, adults included. In addition, left/right change is carried out by a simple reversal, without any additional accessory. A new generation of positioning system is being developed to allow a modular inclination around a bridge axis to obtain many positioning and inclination angles (varying from 0° to 50°). Moreover, this system allows an imaging device (CT or MRI) to be used to verify patient positioning before treatment and to correct patient set up when a variation of organ position occurs.

### Other centres

The technical difficulties and costs involved in moving a proton beam around the patient led to a search for new solutions in patient positioning and movement. The idea to move the patient instead of the beam had been pursued in proton therapy centres at iThemba Labs in South Africa [9] and at the Centre de Protontherapie d'Orsay in France [10,11].

The MPRI robotic system was the first attempt in the USA to use industrial robots for patient positioning in radiotherapy [12]; the commercially available IBA proton therapy systems, installed at the Francis H. Burr Proton Therapy Centre at the Massachusetts General Hospital in Boston as well as at the University of Florida Proton Therapy Centre in Jacksonville, employ custom manufactured robotic-based treatment couches [13].

In Germany, Siemens has developed a robotic positioning system similar in some respects to that of MPRI [14].

## Discussion

The upright or seated position of the patient, obtained with a robotic couch, compared to a fixed proton beam, can reproduce as many entrance possibilities as a proton beam mounted on a gantry. The upright position is more reproducible than the supine/prone position because the distance between the hip-joints and the floor can be more easily controlled and fixed during each treatment session. The skin will be stretched owing to gravity, but this stretching will be approximately the same each time throughout the radiotherapy course unless an extreme loss of weight takes place. Vertical or oblique positioning is compatible with immobilization devices commonly used in radiotherapy. Up to now the position accuracy seems limited due to the anatomical data acquisition by means of CT or MRI scanners which both require horizontal (prone or supine) patient positioning. Robotics arms can position the patient in many different ways, however, while the gantries used in proton therapy allow for many beam incidences, the ample theoretical possibilities of movement of the robotic couch arms are relatively limited by the fixed positioning requirements of CT (MRI) scanners. Future innovations should involve a wider range of movements of the couch and the possibility to acquire tomographic images in the treatment setup position of the patient which could also be non-horizontal.

Many systematic literature reviews regarding cost-effectiveness suggest that at the moment there is no clear evidence in favour or against proton radiotherapy as opposed to conventional photon radiotherapy. Some Authors suggest a net advantage of proton therapy for a limited number of tumour sites, such as uveal melanomas and others ocular tumours, skull base chordomas and chondrosarcomas, medulloblastoma in paediatric patients [15,16]. For other pathologies such as breast, prostate, head-and neck tumours, similar evidence has been reported for selected patient sub-groups [17-19]. This unclear evidence is based on the fact the proton-therapy facilities with gantry are more expensive compared to traditional radiotherapy centres. Thus, the cost effectiveness for each individual patient is outweighed by the clinical advantages of proton radiotherapy.

Due to the automatic positioning with the specific robots, the cost of the proton therapy facility can almost be halved, making it cost effective for the patient. Moreover, adequate imaging devices for daily check positioning could reduce the time of patient set up as well as the overall treatment time, and thus permit more patients to undergo therapy. Furthermore, the building costs of proton therapy facilities decreases when gantries are not included in the cost calculation, associated with significantly increased shielding, installations and running costs [1]. This would then allow an increase in the number of treatment rooms.

The main drawback is the cost of proton therapy facilities which in turn limits the number of patients undergoing this new modality of treatment. Therefore, the use of automatic positioning could bring down the costs and lead to an immediate and more widespread use of proton therapy. New automatic devices are necessitated to improve again the actual technology.

## Conclusions

A cost reduction in building proton therapy facilities equipped with robotic systems for patient positioning instead of rotating gantries, is expected to reveal more clearly the clinical advantage of proton versus photon therapy supported by planning studies demonstrating improved dose distribution.

## Competing interests

The authors declare that they have no competing interests.

## Authors' contributions

SD, VD and MP searched the literature for relevant contributions and helped to draft the manuscript. LS, MDA and MB conceived of the review, designed it and refined the draft version of the manuscript. All authors read and approved the final manuscript.

## References

[B1] Goitein M Jermann M The relative costs of proton and x-ray radiation therapy Clin Oncol 2003 15 S37 S50 10.1053/clon.2002.0174 12602563

[B2] Flanz J Technology for proton Therapy The Cancer Journal 2009 15 292 297 10.1097/PPO.0b013e3181b11dd0 19672145

[B3] Goiten M Trials and tribulations in charged particle radiotherapy Rad Oncol 2009 in press 10.1016/j.radonc.2009.06.012 19581014

[B4] Smith AR Vision 20/20: Proton therapy Med Phys 2009 36 556 568 10.1118/1.3058485 19291995

[B5] Langen KM Jones DTL Organ motion and its management Int J Radiat Oncol 2001 50 265 278 10.1016/S0360-3016(01)01453-5 11316572

[B6] Katuin JE Schreuder AN Starks WM Doskow J Duggan J, Morgan I The use of industrial robots for high precision patient positioning Conference on the Application of Accelerators in Research and Industry" (CAARI 2002): Proceedings of the 17th International Conference on the Application of Accelerators in Research and Industry, 12-16 November 1998, Denton, TX 1998 American Institute of Physics: Melville, New York

[B7] Bolsi A Lomax AJ Pedroni E Goitein G Hug E Experiences at the Paul Scherrer Institute with a remote patient positioning procedure for high-throughput proton radiation therapy Int J Radiat Oncol Biol Phy 2008 71 1581 90 10.1016/j.ijrobp.2008.02.079 18640501

[B8] Sevret P Magnè N Chargari C An orginal contention system for hadrontherapy Cancer/Radiothérapie 2009 13 161 163 10.1016/j.canrad.2009.01.007 19297227

[B9] Schreuder AN Duggan J, Morgan I The non-orthogonal fixed beam arrangement for the second proton therapy facility at the national accelerator centre Conference on the Application of Accelerators in Research and Industry" (CAARI 1998): Proceedings of the 15th International Conference on the Application of Accelerators in Research and Industry, 4-7 November 1998 1998 American Institute of Physics: Melville, New York 963 966

[B10] Ferrand R Sisterson J Patient positioning system at CPO: test and commissioning Particle Therapy". Cooperative Group Meeting (PTCOG XXVI): Abstracts of the XXVI PTCOG Meeting 1997 Harvard Cyclotron Laboratory: Boston, MA 16 17

[B11] Mazal A Robots in high precision patient positioning for conformal radiotherapy Proceedings of World Congress on Medical Physics and Biomedical Engineering, Medical and Biological Engineering and Computing (NICE '97), 14-19 September 1997 35 Nice, France 824

[B12] Allgower EC Schreuder AN Farr JB Mascia AE Experiences with an application of industrial robotics for accurate patient positioning in proton radiotherapy Int J Med Robotics Comput Assist Surg 2007 3 72 81 10.1002/rcs.128 17441029

[B13] Meggiolaro MA Dubowsky S Mavroidis C Geometric and elastic error calibration of a high accuracy patient positioning system Mechanism Machine Theory 2002 40 415 427 10.1016/j.mechmachtheory.2004.07.013

[B14] Eickhoff H Haberer T Status report of the HICAT/HIT Project GSI Scientific Report HICAT-HD-01 2004 http://www.gsi.de/informationen/wti/library/scientificreport2004/PAPERS/HICAT-HD-01.pdf

[B15] Lodge M Pijls-Johannesma M Stirk L Munro AJ De Ruysscher D Jefferson T A systematic literature review of the clinical and cost-effectiveness of hadron therapy in cancer Radiother Oncol 2007 83 110 122 10.1016/j.radonc.2007.04.007 17502116

[B16] Lundkvist J Ekman M Ericsson SR Jönsson B Glimelius B Cost-effectiveness of proton radiation in the treatment of childhood medulloblastoma Cancer 2005 103 793 801 10.1002/cncr.20844 15637691

[B17] Lundkvist J Ekman M Ericsson SR Isacsson U Jönsson B Glimelius B Economic evaluation of proton radiation therapy in the treatment of breast cancer Radiother Oncol 2005 75 179 185 10.1016/j.radonc.2005.03.006 15885828

[B18] Lundkvist J Ekman M Ericsson SR Jönsson B Glimelius B Proton therapy of cancer: potential clinical advantages and cost-effectiveness Acta Oncol 2005 44 850 861 10.1080/02841860500341157 16332592

[B19] Glimelius B Ask A Bjelkengren G Björk-Eriksson T Blomquist E Johansson B Karlsson M Zackrisson B Number of patients potentially eligible for proton therapy Acta Oncol 2005 44 836 849 10.1080/02841860500361049 16332591

